# Does Chemotherapy Have an Effect on the Treatment Success of Children and Adolescents with Unresectable Hepatocellular Carcinoma? Findings from the German Liver Tumour Registry

**DOI:** 10.3390/cancers17152444

**Published:** 2025-07-23

**Authors:** Mark Rassner, Beate Häberle, Rebecca Maxwell, Julia von Frowein, Roland Kappler, Michael Rassner, Christian Vokuhl, Dietrich von Schweinitz, Irene Schmid

**Affiliations:** 1 Department of Pediatrics, Dr. von Hauner Children’s Hospital, University Hospital, LMU Munich, Lindwurmstraße 4, 80337 Munich, Germany; mark.rassner@med.uni-muenchen.de (M.R.); rebecca.maxwell@med.uni-muenchen.de (R.M.); julia.frowein@med.uni-muenchen.de (J.v.F.); 2 Department of Pediatric Surgery, Dr. von Hauner Children’s Hospital, University Hospital, LMU Munich, 80337 Munich, Germany; beate.haeberle@med.uni-muenchen.de (B.H.); roland.kappler@med.uni-muenchen.de (R.K.); dietrich.schweinitz@med.uni-muenchen.de (D.v.S.); 3 Faculty of Medicine, Clinic for Internal Medicine I, Haematology, Oncology and Stem Cell Transplantation, University Medical Center Freiburg, 79106 Freiburg, Germany; michael.rassner@uniklinik-freiburg.de; 4 Section of Pediatric Pathology, Institute of Pathology, University Hospital Bonn, Venusberg-Campus 1, 53127 Bonn, Germany; christian.vokuhl@ukbonn.de

**Keywords:** hepatocellular carcinoma, unresectable, response to chemotherapy

## Abstract

**Background**: Paediatric hepatocellular carcinoma (HCC), including its fibrolamellar variant (FLC), is a rare malignancy with distinct biological behaviour and limited therapeutic options. While complete surgical resection is a key determinant of survival, many patients present with unresectable tumours at diagnosis. The role of neoadjuvant chemotherapy in improving resectability, particularly in histologically distinct subtypes, remains inconclusive. **Methods**: We retrospectively analysed 43 patients (<18 years) with histologically confirmed conventional HCC (cHCC, *n* = 27) or FLC (*n* = 16) enrolled in the German Pediatric Liver Tumour Registry. We assessed clinical characteristics, treatment response, surgical outcomes, and survival. Special focus was placed on the impact of neoadjuvant chemotherapy in initially unresectable tumours. **Results**: FLC and cHCC exhibited significant differences in clinical presentation, such as age of presentation, AFP elevation, or presence of underlying liver disease. Although overall survival did not significantly differ between groups, cHCC tumours showed a markedly higher response to chemotherapy (62.5% partial remission vs. 0% in FLC). Complete resection (R0) was achieved in 77% of all patients and was the strongest predictor of survival. Importantly, a subset of cHCC patients who initially had unresectable tumours became eligible for curative surgery following neoadjuvant chemotherapy. Notably, delayed resection after chemotherapy led to outcomes comparable to those with upfront surgery, whereas progression during chemotherapy was associated with a universally poor prognosis. **Conclusions**: This study supports upfront resection as the preferred strategy in paediatric HCC and FLC whenever feasible. In cHCC, neoadjuvant chemotherapy demonstrated a favourable response profile and contributed to secondary resectability in a subset of initially unresectable cases, supporting a potential role within a multimodal treatment approach. In contrast, FLC showed limited responsiveness to current systemic therapies. These findings emphasise the importance of histology-specific strategies and highlight the ongoing need for more effective systemic options, particularly for unresectable FLC.

## Simple Summary

Paediatric hepatocellular carcinoma (HCC), including its fibrolamellar variant (FLC), is a rare malignancy often diagnosed at an advanced, unresectable stage. In this retrospective analysis of 43 patients, conventional HCC (cHCC) and FLC demonstrated distinct clinical features and treatment responses. Complete surgical resection remained the strongest predictor of survival in both groups. Notably, cHCC showed a meaningful response to neoadjuvant chemotherapy, which enabled secondary resection in several initially unresectable cases, suggesting that chemotherapy may be a valuable component of treatment in selected patients. In contrast, FLC exhibited minimal chemosensitivity. These findings support upfront surgery whenever feasible and highlight the importance of histology-specific treatment approaches, particularly the need for more effective systemic therapies for FLC.

## 1. Introduction

Primary malignant tumours of the liver are rare and account for only approximately 1% of all paediatric cancer patients [1] or 1.6 cases per million children (0–14 years) [2,3,4]. In contrast to hepatoblastoma, hepatocellular carcinoma (HCC) is even less common, with its incidence increasing with age. Etiologic factors can be detected only in a fraction of paediatric HCC patients, such as in areas with high prevalence of chronic hepatitis B virus (HBV) infection [5], or in the case of underlying chronic liver disease (e.g., glycogen storage disease type III, tyrosinemia type I) [6], reflecting a different pathogenesis [7] than in adults. Moreover, while fibrolamellar carcinoma (FLC) is extremely rare in adult HCC cases (~1%) [8], it is significantly more common in the paediatric population [9,10]. Differences between FLC and conventional hepatocellular carcinoma (cHCC) are evident in their biological underpinnings, patterns of locoregional and distant spread at diagnosis, and overall clinical course [9,11,12,13], e.g., absence of elevated α-fetoprotein (AFP) levels [14,15] and lack of underlying liver disease [16] often delay diagnosis of FLC, and characteristic fusions between *PRKACA* and *DNAJB1* were identified as oncogenic driver in FLC, but not in cHCC [12,17].

Analyses indicate that the 5-year overall survival rate in paediatric patients with HCC has significantly improved over the past decades—from less than 25% in the 1990s [2,18,19] up to around 40–50% by the 2010s [13,20,21]. Currently, patients with resectable HCC achieve a 3-year survival of approx. 80%, while patients with non-resectable HCC have an unacceptably low survival of only 10–20% [4].

Therapy concepts in the treatment of HCC are usually multimodal [4], including surgery, chemotherapeutic agents, targeted therapies, and interventional radiology. Achievement of complete resection has been identified as the fundamental prognostic determinator, irrespective of the individual treatment approach [18,22,23,24], and upfront surgery, whenever feasible, has been advised in major international treatment studies, such as the SIOPEL 3 trial. However, a high prevalence of locally advanced tumour stages, often accompanied by metastases at the time of diagnosis, challenges resection by partial hepatectomy in a majority of patient cases [25]. Contributing to its oftentimes intractable behaviour, presentation with large tumour size at diagnosis [9], and high rates of vascular invasion and nodal spread [11] further complicate surgical resection of FLC tumours.

Thus, on the one hand, liver transplantation (OLT) has increasingly been performed in unresectable paediatric HCC with promising OS rates [22,26,27,28,29], which resulted in a paradigm change for OLT beyond the Milan criteria in children. On the other hand, other treatment modalities have hence primarily been employed to try to achieve secondary resectability, by either partial hepatectomy or by OLT.

In adults, HCC represents a highly chemoresistant entity, and, thus, official guidelines do not recommend application of chemotherapy as first-line therapy [30,31]. In contrast, in children and adolescents, a chemotherapy response rate of almost 50% was demonstrated by the first International Society of Pediatric Oncology Liver Tumour Study (SIOPEL-1 study, 1990–1994) [2]. Whereas, unfortunately, only low complete tumour resection rates were achieved in this and subsequent treatment trials [32], secondary resectability of primarily unresectable tumours could be demonstrated by the combination of chemotherapy and sorafenib in a small case series [33]. Since paediatric HCC is so rare—with a distinct variety of treatment concepts between studies—conclusive information about clear benefit from neoadjuvant chemotherapy with respect to resectability still remains scarce [20,22,23].

In the present study, patients < 18 years of age with unresectable cHCC or FLC were retrospectively examined to determine whether neoadjuvant chemotherapy can lead to resection of tumours/metastases and, thus, achieve a chance of cure. In addition, to further clarify indications for chemotherapy, we aimed to evaluate differences between tumours with cHCC or FLC histology with regard to response to chemotherapy. When examining patients without primary resection, we found a good response to chemotherapy in patients with cHCC tumours, but not for FLC. Resectability either improved or became possible in a considerable fraction of patients who responded to chemotherapy. In support of aggressive surgical strategies for children with cHCC or FLC, primary resection at diagnosis should be pursued whenever feasible. Our findings further suggest that chemotherapy benefits patients with initially unresectable cHCC, while highlighting the urgent need for novel chemotherapy or other systemic treatment approaches in FLC.

## 2. Materials and Methods

Patients from the German Society for Pediatric Oncology and Haematology (GPOH) centres in Germany, Austria, and Switzerland were included in this study through the ongoing Liver Tumour Registry (LTR), which began enrolment of paediatric liver patients on 17 January 2011. Latest recruitment of included patients was on 16 February 2022. Ethical approval for the LTR was granted by the Ethics Committee at Ludwig-Maximilians-University (LMU) in Munich, where data are centrally collected and managed. Written informed consent was obtained at the time of enrolment. Clinical data were extracted retrospectively for the purposes of this analysis.

Eligible patients included in the present analysis were younger than 18 years and had a reference pathologist histologically confirmed conventional hepatocellular carcinoma (cHCC) or fibrolamellar carcinoma (FLC). Demographic, clinical, radiologic, and treatment data were obtained from the respective datasets. Deep analysis was performed on non-resectable cases of cHCC or FLC at diagnosis, defined as either (1) locally advanced disease with contraindications to primary surgical resection (partial hepatectomy) or liver transplantation (OLT), and/or (2) the presence of distant metastases not amenable to complete surgical removal. Primary surgery was indicated at diagnosis, whenever feasible, for all patients according to the standard of care. If unresectable, initial therapy at diagnosis varied by date of diagnosis and applicable institutional protocol at that period in time, but typically included chemotherapy regimens based on cisplatin and doxorubicin (PLADO). Treatment response was evaluated with abdominal MRI and/or sonography, and thoracic CT scans after 2 and 4 cycles of chemotherapy. Patients achieving resectability underwent surgical resection with curative intent. Follow-up time was at least 12 months for all patients, unless death occurred prior to this point.

The extent of resection was categorised as R0 (microscopically complete), R1 (microscopically incomplete), or R2 (macroscopically incomplete) based on operative and histopathologic reports. Outcome measures included overall survival (OS) and state of remission. OS was defined as the time from diagnosis to death from any cause or last follow-up. Complete remission was defined as the disappearance of all detectable tumour lesions at the completion of treatment. Relapse was defined as the reappearance of tumour lesions on imaging after a prior complete remission, while progression was defined as tumour growth in patients who had not achieved remission.

Due to the small number of patients, statistical analysis was descriptive and exploratory. Numeric variables were compared using the Wilcoxon rank-sum test; categorical variables were compared using Fisher’s exact test. Kaplan–Meier curves were used to assess OS, and hazard ratios (HRs) with 95% confidence intervals (CIs) were calculated using the Mantel–Haenszel method. All statistical analyses were performed using GraphPad Prism Version 10.4.1. A significance threshold of *p* < 0.05 was used for all comparisons.

## 3. Results

### 3.1. Patient Demographics and Tumour Staging

A total of 43 paediatric patients were treated for HCC in our cohort (see Table 1), including 27 (60.0%) cHCCs and 16 (35.6%) FLCs; in accordance with other studies [13], mean age at diagnosis differed significantly between children presenting with cHCC (143.6 months) or FLC (182.5 months) (*p* = 0.007). Moreover, elevation of α-fetoprotein (AFP) > 100 ng/mL was exclusively observed in children with cHCC, where 74.1% of documented cases exceeded this threshold, in contrast to FLC, where no elevations beyond 100 ng/mL were reported (*p* < 0.001). While underlying liver disease was found in only 18.6% of all patients, all of these presented with cHCC (29.6% of all children with cHCC), and in our patient collective, no case of FLC with prior liver pathology was reported (*p* = 0.013). Children with underlying liver disease were significantly younger at time of diagnosis than patients without (118.0 months vs. 168.8 months, *p* = 0.029). Similarly to other reports [13], we found a tendency for more extensive spread within the liver for cHCC when comparing pretreatment extent of disease (PRETEXT) between cHCC (III/IV 70.4%) to FLC (III/IV 31.8%). In contrast, age did not significantly correlate with PRETEXT group (mean age PRETEXT I 150.8 months, PRETEXT IV 139.5 months) (*p* > 0.999), nor was there an apparent correlation between underlying liver disease and pretreatment extent of disease (*p* = 0.447). Albeit not statistically significant in our collective, patients with FLC tumours were more likely to present with metastasis than patients who had cHCC (50.0% vs. 33.3%), as previously reported [13]. There was, however, a significant correlation between age and metastasis—while mean age of patients without metastasis at time of diagnosis was 149.0 months, patients with metastasis were significantly older (172.0 months) (*p* = 0.043). Furthermore, there was a clear tendency for higher metastasis rate in patients without prior liver pathology than in patients with underlying disease (50.0% vs. 12.5%) (*p* = 0.107). Similarly, despite small subgroup number limiting statistical significance, higher PRETEXT stage seemed to correlate with higher metastasis rate (16.7% for PRETEXT I vs. 66.7% for PRETEXT IV).

### 3.2. Treatment

Biopsy-related confirmation of diagnosis (with central review) was achieved in the majority of cases (79.1%), and there was no significant difference between histologic or age groups (Table 2). However, biopsy was performed in a significantly smaller fraction of patients with underlying liver disease (37.5%) compared with those without prior liver morbidity (88.2%) (*p* = 0.0049).

A total of 34 patients (79.1%) received chemotherapy, and 29 patients (67.4%) received treatment with a protein kinase inhibitor (predominantly Sorafenib); thus, many patients received both treatments. Neoadjuvant therapy was administered to 21 chemotherapy recipients and 16 kinase inhibitor recipients. Moreover, 21 and 16 patients, respectively, were treated in a neoadjuvant setting. The rate of chemotherapy or kinase inhibitor treatment did not differ between histological groups, but patients receiving Sorafenib tended to be older than those who did not (*p* = 0.0237). Moreover, patients with prior liver disease were less likely to receive either chemo- or small molecule therapy at any time point (*p* < 0.01). Patients without metastases were less likely to receive Sorafenib in our patient cohort (*p* = 0.0012), but there was no significant difference with regard to chemotherapy.

A total of 36 out of 43 patients (83.7%) had either a primary or a secondary tumour operation. Moreover, 29 of these children (67.4%) underwent partial hepatectomy, while orthotopic liver transplantation (OLT) was performed in 7 cases (16.3%). Complete resection was achieved with almost all surgical interventions (33/36; 91.7%). Interestingly, in our patient collective, the complete surgical resection rate was not inferior in cases with operation after neoadjuvant therapy; all 16 patients operated after prior chemotherapy had no gross or microscopic residual disease (R0), while this rate was slightly lower for children with upfront resection at diagnosis (17/20; 85%). Extent of resection did not differ between histological groups (*p* = 0.4596), nor was there any significant difference in rate or mode of surgery. However, OLT was more commonly performed in patients with cHCC, comparable to previous reports [13]. There was no difference in surgical outcome with regard to age.

Children with underlying liver disease had a significantly higher rate of OLT than those patients without (50% vs. 8.8%; *p* = 0.0085). Overall resection rate tended to be smaller in highly advanced tumours (50.0% in PRETEXT IV tumours vs. 83.3% with PRETEXT I), and OLT rate was higher with increasing PRETEXT stage.

Patients with distant metastasis were significantly less likely to have surgical resection of their primary tumour (64.7% vs. 96.2%; *p* = 0.0106), and only one out of seven OLTs was performed among patients with metastasis at diagnosis. Similarly, almost all patients without distant metastasis at diagnosis achieved complete remission (96.2%), in contrast to patients with metastatic disease, in which complete resection was accomplished in only 47.1% of patients (*p* = 0.0003).

### 3.3. Outcome and Overall Survival in Patient Subgroups

**Figure 1:**
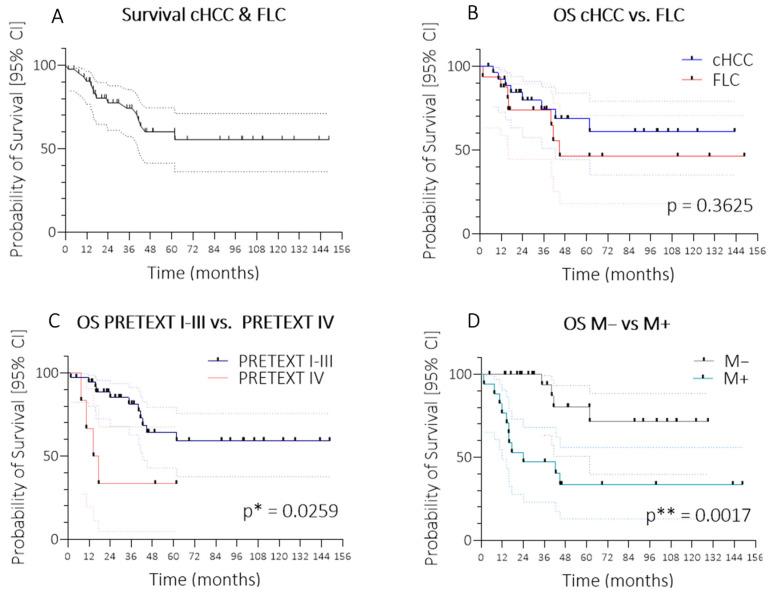
Overall survival and survival in patient subgroups. Kaplan–Meier curves showing: (**A**) overall survival (OS) in the entire cohort of patients with hepatocellular carcinoma (HCC); (**B**) OS stratified by histological subtype: conventional HCC (cHCC) versus fibrolamellar carcinoma (FLC); (**C**) OS stratified by PRETEXT group: I–III versus IV; and (**D**) OS stratified by metastatic status: M− (no metastases) versus M+ (presence of metastases). For statistical significance, *p* * < 0.05; *p* ** < 0.01. Abbreviations: cHCC, conventional hepatocellular carcinoma; FLC, fibrolamellar carcinoma; OS, overall survival; PRETEXT, pretreatment extent of disease; M, metastases.

In total, 15 children died within the observation period (Figure 1A). Long-term survival comprised 55.5%. Interestingly, with the exception of a single patient, almost all events of death occurred within 60 months after initial diagnosis, without any additional long-term deaths. Due to low patient numbers, there was no significant difference in mortality rate between cHCC and FLC (*p* = 0.3625). In contrast to recent reports [13], mortality rates in children with cHCC did not exceed FLC at early time points after diagnosis (Figure 1B), and, as a matter of fact, overall survival was even higher 60 months after diagnosis (68.6% in cHCC vs. 46.2% in FLC), despite comparable tumour stages (local extents or metastasis rates) in cHCC and FLC (Table 1). Given its association with mortality rates in hepatocellular neoplasms, patient groups with low (<100 ng/mL (22 patients)) or high (≥100 ng/mL (20 patients)) AFP concentrations (recorded at diagnosis) were also evaluated. Although there was a trend for inferior long-term survival in patients with low AFP expression, this difference was not significant in our patient cohort (*p* = 0.5621). AFP was not found to be increased in any patient with FLC tumour; however, even when mortality was compared within cHCC, specifically, rates did not differ significantly between the AFP low or high groups, either (*p* = 0.7863). Notably, mortality rates were still higher in the most advanced PRETEXT IV group compared to lower stages (*p* = 0.0259; Figure 1C). Median survival was only 16.0 months in children with locally advanced tumours, while long-term survival was 59.3% in all other patients. Presence of distant metastases at diagnosis (39.5% of all patients with cHCC or FLC) was associated with substantially worse long-term survival (33.6% compared to 71.4% in patients without metastases (*p* = 0.0017)), and median survival was approx. 24.0 months in these children (Figure 1D).

**Figure 2:**
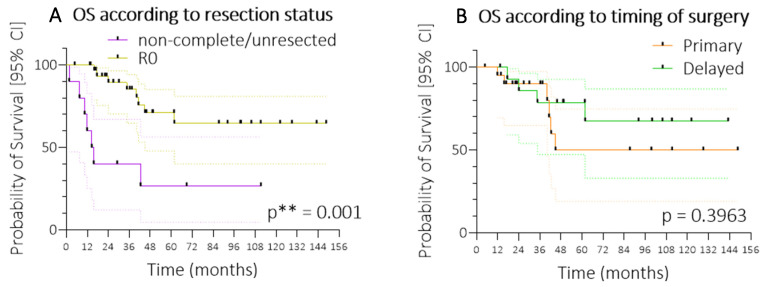
Survival according to resection status and timing of surgery. Kaplan–Meier curves showing (**A**) overall survival (OS) stratified by resection status—non-complete or unresected versus R0 resection (complete resection with negative margins)—and (**B**) OS stratified by timing of surgery—primary resection versus delayed resection following initial treatment. For statistical significance, *p* ** < 0.01. Abbreviations: OS, overall survival; R0, complete resection with microscopically negative margins.

Similarly, while complete resection of the main tumour mass was achieved in a considerable 33 out of all 43 patients with cHCC or FLC (76.7%), long-term survival in patients with non-resected or incomplete resection was profoundly worse than in patients with complete resection (26.7% vs. 64.5%; *p* = 0.001; Figure 2A). Analogously, median survival was only 15.1 months in patients without R0-resection. Among patients with surgical resection, the time point of intervention was not associated with outcome (*p* = 0.3963) (Figure 2B): Long-term survival in patients with secondary or delayed resection (that is, after neoadjuvant therapy) did not significantly differ from those with resection at diagnosis.

In summary, increased risk for death (hazard ratio) was found for patients with locally advanced tumours (PRETEXT IV), distant metastasis, and, particularly, in cases with unresected tumours (Table 3).

### 3.4. Secondary Resectability of Initially Unresectable Tumours

Given the critical impact of complete tumour resection on mortality and long-term survival, upfront surgical resection—whenever feasible—has been a cornerstone of previous therapeutic strategies [2]. This recommendation has been consistently upheld in patients enrolled in the Liver Tumour Registry, underscoring the importance of rigorous assessment of resectability prior to considering preoperative chemotherapy. We thus wondered if, and to what extent, neoadjuvant chemotherapy, in addition to advances in operative techniques, may have contributed to high resection rates. It might, at least in part, be responsible for converting primary unresectable tumours into ones that can be treated via partial hepatectomy or orthotopic liver transplantation, or for enabling a partial hepatectomy where previously an orthotopic liver transplant would have been required.

When compared to each other, the response to chemotherapy differed substantially between tumours with cHCC vs. FLC histology. Whereas partial remission was achieved in at least 10/16 (62.5%) patients with cHCC (stable disease, SD (3), progressive disease, PD (2), n.a. (1)), not a single patient with FLC demonstrated significant reduction in both primary and metastatic tumours (SD (3), PD (1), n.a. (1)).

Similarly, among ten patients with cHCC who received Sorafenib, seven patients had a partial response (PR, 70%). In contrast, partial remission was lower for patients without Sorafenib (3/6 (50%)); however, all of these 16 patients also received chemotherapy. All evaluable patients with FLC tumours received Sorafenib, but none displayed partial remission (s. above).

**Figure 3:**
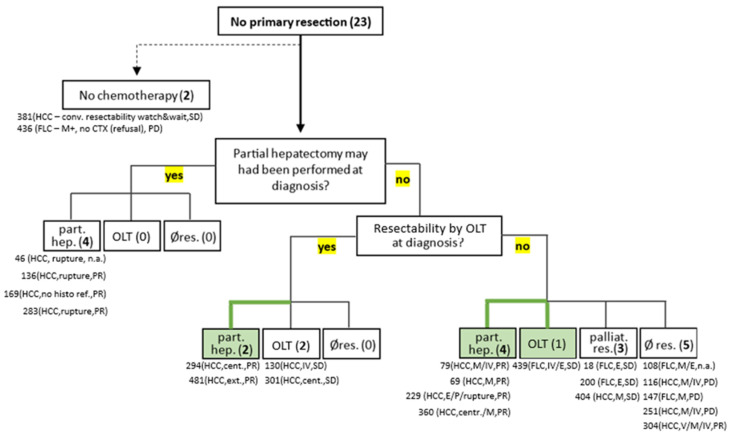
Flowchart of initial resectability assessment and final surgical procedures after chemotherapy. Flow diagram illustrating the resectability assessment in patients who did not undergo primary resection. Two patients did not receive chemotherapy and were excluded from further evaluation. The remaining patients were evaluated for initial resectability, either by partial hepatectomy or, if not feasible, by OLT, to assess whether surgical intervention may have been possible at diagnosis. The final surgical procedure following chemotherapy is indicated for each group. Below treatment groups, patients are listed by UPN, histology, reason for neoadjuvant therapy, and response to chemotherapy. Abbreviations: conv., conventional; SD, stable disease; PD, progressive disease; PR, partial response; M, metastasis; E, extrahepatic disease; part. hep., partial hepatectomy; cent., central; ext., extent; OLT, orthotopic liver transplantation; Ø res., no resection; palliat. res., palliative resection.

First, focusing on all cases without resection at diagnosis (primary resection; Figure 3, Table 4), two patients without chemotherapy at any time point were identified. One patient (Unique patient number (UPN) 381) suffers from severe underlying disease (Adenosine kinase deficiency) with profound disease burden and was, thus, not considered eligible for operation or chemotherapy by the health care provider. Fortunately, stable disease (SD) was recorded at the last follow-up, 29 months after initial diagnosis. Presenting with distant metastases at diagnosis, the second patient (UPN 436) had an indication for neoadjuvant therapy. While the primary tumour may have been resected initially, chemotherapy was refused by the patient’s family, and the tumour progressed and became unresectable, despite later small-molecule and antibody therapy. Selective internal radiation (SIRT) was then performed, and the patient remains alive >68 mo. after diagnosis.

Since the benefits of neoadjuvant chemotherapy become particularly clear when resectability either improves or has only just been achieved (by response to therapy), we reasoned that causes or indications for neoadjuvant therapy along with the actual initial state of resectability need to be thoroughly and critically evaluated in order to not falsely overestimate chemotherapeutic effects, especially in cases, in which tumours might, in fact, have been resected by partial hepatectomy at diagnosis. For this purpose, patients were further grouped according to the resectability of their tumours at the time of diagnosis.

Firstly, four patients (UPN 46, 136, 169, and 283), all with cHCC histology, were identified, in whom partial hepatectomy could have been performed at the time point of diagnosis. However, three of them manifested by tumour rupture, while the fourth was first misdiagnosed as hepatoblastoma. Interestingly, partial remission was achieved in three of these children, while response was not evaluable in the last patient due to surgical intervention shortly after the start of the neoadjuvant therapy. All four patients achieved complete resection of their tumours (R0). Moreover, there were no patients who were clearly feasible for partial hepatectomy at diagnosis and had to undergo liver transplantation after chemotherapy or were then left unresected.

Next, a total of four patients were identified, who had either central (UPN 294, 301) or locally advanced tumours (UPN 130, 481), who were not eligible for partial hepatectomy, but for resection by orthotopic liver transplantation (without contraindications) at diagnosis. Tumours of these patients shared cHCC histology and, fortunately, partial remission through chemotherapy was achieved in two patients, making them amenable for secondary conventional resection, while the remainder showed stable disease and subsequently went through liver transplantation (in addition, HB histology was initially assumed for one of these patients). Comparable to the previous patient group, there were no patients who developed progression under chemotherapy (extrahepatic/metastatic disease), rendering them unresectable during chemotherapy.

Another 13 patients fulfilled clear criteria for neoadjuvant therapy with extrahepatic (5/13; 38.5%) and/or distant metastatic (9/13; 69.2%) disease, as well as additional complicating factors in some of these cases (tumour extent and localization). Five patients with cHCC histology showed partial remission with neoadjuvant chemotherapy. Despite the response, further therapy was discontinued by one patient (UPN 304), who ultimately died from the disease 7.5 months after diagnosis, having never had a surgical resection. All four other patients (UPN 69, 79, 229, and 360) became eligible for partial hepatectomy and achieved complete histopathological resection.

Four patients with extrahepatic or metastatic disease (UPN 18, 200, 404, and 439), three with FLC and one with cHCC subtype, showed stable disease with chemotherapy, rendering these cases palliative by oncologic definition. Strikingly, surgical resection with complete histopathological remission of tumours, including resection of distant metastases (UPN 404) and transdiaphragmal tumour bulk (UPN 18), was, nevertheless, achieved in all four patients. Moreover, no death has been reported among these patients. However, in two of these children, extrahepatic manifestation (lymph nodes) had been determined after operation, including one case, where liver transplantation was indicated and performed (PRETEXT IV tumour (UPN 439)).

Finally, despite neoadjuvant chemotherapy, tumours were not amenable to resection in five children, who all succumbed to disease. In addition to patient UPN 304 with cHCC histology, who, in spite of partial remission, discontinued therapy (see above), response to chemotherapy was not evaluable due to rapid death in another child with FLC tumour (UPN 108). Three other patients, two with cHCC (UPN 116, 251) and one with FLC (UPN 147), unfortunately, developed tumour progression during chemotherapy. Presenting with a locally advanced tumour requiring liver transplantation, OLT was contraindicated due to the progress of metastases (UPN 116, 251), although both the main tumour and metastases were still considered principally resectable, even after progress in at least one patient case (UPN 116).

In summary, in contrast to FLC, the chemotherapy response rate was high in patients with cHCC histology. While, on closer inspection, not all tumours that were resected after chemotherapy were, in fact, unresectable at time point of diagnosis (either by partial hepatectomy or by orthotopic liver transplantation), there were no cases, in which chemotherapy led to inferior surgical resectability, rather it appeared to make some tumours amenable to partial hepatectomy or liver transplantation. Best response in patients with FLC was SD, but nevertheless, complete resection, including extrahepatic manifestation, was achieved in these tumours, enabling long-term survival. Unfortunately, all patients with unresectable and progressive disease after chemotherapy died of the disease.

Similarly to chemotherapy, response rates to Sorafenib were high in patients with cHCC tumours (7/10; 70%), in contrast to those with FLC tumours (0/5; 0% (1 n.a.); however, only one patient (FLC) did not receive additional chemotherapy, impeding statistical conclusions about Sorafenib effects.

## 4. Discussion

In our study, we retrospectively evaluated patient characteristics and treatment outcomes of 43 children treated for either cHCC or FLT.

While the distribution of cHCC and FLC histology tumours was comparable to other studies, profound differences between these two were determined, in accordance with other studies [13]. For instance, the mean age at diagnosis was significantly higher in children with FLC, and, in contrast to cHCC tumours, there were no patients with AFP elevations above 100 ng/mL. Underlying liver disease resulted in cHCC tumours, exclusively. Moreover, cHCC tumours had higher pretreatment extent of disease, whereas patients with FLC tumours were more likely to present with metastasis. In conjunction with the detection of a recurrent, potentially pathognomonic fusion event in FLC, but not in cHCC (*DNAJB1-PRKACA*) [12], and in accordance with previous reports [34,35,36], all of these observations suggest that cHCC and FLC tumours do represent different entities with different biological background, manifestation, and clinical course, and thus, require distinct therapeutic handling (s. below).

Age was lower for children with underlying liver disease, and older children were more likely to present with metastasis at diagnosis. Consequently, the metastasis rate was lower for patients in the context of pre-existing liver pathology. In addition to a divergent biomolecular background, regular routine screening and earlier detection of lesions in patients with chronic liver disease may contribute to these findings.

Overall, compared to extremely poor prognosis in the 1990s [2,18,19] (5-year OS ~25%), a significant improve in survival rates up to around 40–50% by the 2010s has been reported in some studies [13,20,21]. Similarly, long-term survival was 55.5% (95% CI: 36.2–71.1%) in our patient collective (median survival was not reached).

Historically, several predictors of mortality in paediatric HCC have been identified, including local tumour extent, involvement of major vascular structures, multifocality, metastatic disease, elevated AFP concentration, and, importantly, absence of surgical resection [13,37]. Since all patients with resectable tumours were subjected to immediate resection at diagnosis, and the remainder, unfortunately, were not successfully converted resectable through neoadjuvant chemotherapy, upfront surgery was associated with better patient outcome, in earlier studies [18]. More recently, higher mortality rates for tumours resected after chemotherapy were reported by Short et al. [13] in a univariable Cox Model for Death. In accordance, in our study, high local tumour extent (PRETEXT stage IV) was also associated with inferior long-term survival in our patient collective, and, similarly, the prognosis of children with metastasized disease was significantly worse than in cases without. Interestingly, no significant difference in long-term survival was found for comparison between AFP low or AFP high tumours, even when only cHCC tumours were considered, given that there were no cases of AFP elevation in FLC carcinomas. Complete surgical resection was still identified to represent the main therapeutic determinant for survival, although the time point of intervention (primary vs. secondary after chemotherapy) was not significantly associated with patient outcome among resected patient cases.

Overall, a considerable R0 resection of 77.8% (35/45) was achieved in our patients, and this is likely the major reason for the improved survival in our patient collective. Several factors may have contributed to this positive development.

First, improved survival and resection rates may result from screening and strict monitoring, introduced in children with underlying liver disease over the last few decades [6,13,23]. However, only a small fraction of all patients suffered from known underlying liver pathologies in our patient cohort, and, importantly, resection rates did not differ significantly between children with or without predisposing liver comorbidity (75% vs. 77.8%).

Secondly, increasing resection rates have been attributed to advances in surgical techniques [6,23] and a “more aggressive” approach in children, compared to adult patients [20] (despite comparable rates of metastasis and more advanced stages in disease).

Third, orthotopic liver transplantation has become a cornerstone in the treatment of hepatic carcinomas not amenable to partial hepatectomy, and a series of studies has continuously demonstrated good transplant and excellent survival rate outcomes in children, including cases with or without chronic liver disease and patients who exceeded the Milan criteria [6,22,27,38,39,40]. Moreover, when evaluating 73 cases of HCC, Moreno et al. reported a “treatment centre effect” [41], according to which survival rates were significantly better when children were treated at liver transplant hospitals compared to those seen at other hospitals (5-year OS 46.3% vs. 17.5%).

Lastly, when we examined the effects of chemotherapy on secondary resectability in cases of unresectable tumours, clear benefits of neoadjuvant chemotherapy were found. Improved resectability through chemotherapy is well-established for hepatoblastoma, which is highly chemo-sensitive and in which up to 80% of tumours that are unresectable or metastatic at diagnosis do eventually become amenable to surgery after initiation of chemotherapy [37]. In case of hepatocellular carcinoma, however, substantial differences between tumours with cHCC or FLC histology also manifest in response to chemotherapy and, thus, secondary resectability. Differing long-term survival has been a matter of debate for several years, and while some studies found better survival for patients with FLC tumours [6,10,42], equal long-term outcomes were reported in others [7,13,20,21,43,44]. Due to diverse diagnostic, exact surgical, and chemotherapeutic approaches throughout these previous studies, a comparison of patient outcomes is challenging. In our study, no significant difference in long-term survival was found between these two histologic groups. However, while FLC turned out chemoresistant in our patient cohort, response to chemotherapy was high in the cHCC group, where two-thirds of all patients showed partial remission through chemotherapy. On close inspection and comparison of resectability before and after the onset of chemotherapy, some of the patients with partial remission initially presented with resectable disease. Nevertheless, for cHCC tumours, chemotherapy never resulted in inferior surgical resectability, but instead allowed for partial hepatectomy or liver transplantation in some cases. In contrast, patients with progressive disease after chemotherapy, either with cHCC or FLC tumours, eventually all died from the disease.

In conclusion, in agreement with previous reports, our study suggests different therapeutic approaches based on histologic subtype: whereas high response rates were achieved for tumours with cHCC histology—some of them turning resectable only by chemotherapy—it cannot be excluded that, in some instances, resectability may be lost in case of progressive disease. Thus, while chemotherapy can and should be considered in case of unequivocally unresectable disease for cHCC histology tumours, fast and thorough evaluation for primary surgical resection should be carried out, especially to avoid chemotherapy in cases where none would be necessary. For instance, fast resection should be performed in tumours that manifest after rupture. Some of the patients, included in our evaluations, instead received several cycles of chemotherapy before being subjected to surgery, while adjuvant chemotherapy after the operation may have been performed in these cases instead. Additionally, chemotherapy was initiated by some treatment centres, because hepatoblastoma was assumed, and a central review was requested with a delay. If still performed in cases where resectability is already given (for instance, to improve resectability), response to chemotherapy should be monitored extremely carefully in order to avoid unfortunate progression into unresectable disease. For FLC tumours, in comparison, no partial responses were reported. Nevertheless, complete resection was still achieved in patients with stable disease after chemotherapy, despite extrahepatic disease. And, interestingly, liver transplantation was performed in one of these patients, before spreading into the lymph nodes was histologically confirmed after the operation. These results further support the importance of early aggressive surgical approaches for paediatric hepatocellular carcinomas; even though being classified as hardly or not unresectable, complete resection without response to chemotherapy was still achieved in some instances—in one case, even through liver transplantation despite the spread into the lymph nodes. Consequently, in case of FLC tumours, primary surgery should also be considered in case of metastatic disease, or rapidly reconsidered, if signs of progressive disease during chemotherapy are found for cHCC tumours.

In spite of overall progress in therapy, the outcome of children with malignant hepatocellular neoplasms still lags behind survival rates achieved in other paediatric malignancies [1], necessitating other, potentially more effective therapies, especially in the case of unresectable disease. For instance, promising results were reported with transarterial chemoembolization in a small patient series [45]. In adult patients with unresectable HCC, combination therapies including targeted therapy with checkpoint inhibition (e.g., Durvalumab) or with multiple kinase inhibitors (e.g., Lenvatinib) represent the current standard of care [30], with rapid evolvement of new therapy concepts [23]. Partial response rate was high in patients with cHCC tumours and Sorafenib in our collective (70% vs. 50% in patients without Sorafenib); however, all of these patients concomitantly received chemotherapy, further impeding statistical testing. Other, more recent therapy studies (NCT04634357) explore the application of engineered T-cell receptors (TCRs) targeting complexes expressed on the surface of liver cancer cells. FLC tumours, in addition to proving highly resistant towards chemotherapy, present minimal to no response to targeted therapies that have efficacy in cHCC [11,13]. Irresponsiveness to chemotherapy and small molecule inhibitors can be attributed to a combination of several unique molecular characteristics of FLC tumours: *DNAJB1-PRKACA*, the oncogenic driver fusion in FLC leads to abnormal activation of protein kinase A and reprogrammed cellular signalling [17], resulting in lower effectiveness of standard cytotoxic drugs. The fusion also enforces a less differentiated state [46], potentially further reducing susceptibility to chemotherapeutic agents. Additionally, some studies suggest unique immunosuppressive microenvironments, creating barriers to drug delivery and immune-mediated cytotoxicity [47]. There is, however, global data to support the use of gemcitabine and oxaliplatin [48,49] or other oxaliplatin-based regimens. Several trials were conducted to specifically interrogate efficacy of inhibiting a series of putative molecular targets in unresectable FLC [50,51,52], and observations in adult and paediatric patients suggested occasional efficacy of checkpoint inhibition or immune modulators (i.e., interferon), at least in a subgroup of patients and regardless of PD-L1 expression or high mutational burden [53,54,55,56]. Moreover, targeting the oncogenic driver, the *DNAJB1-PRKACA* fusion transcript in FLC, another current trial (NCT04248569), investigates the efficacy of a peptide vaccine combined with checkpoint inhibition, and promising first results have been reported [57].

Thanks to the ongoing efforts in new therapy studies, there is hope that patients with unresectable tumours will soon have access to improved treatment options. This could help enhance the prognosis for children with HCC, bringing outcomes closer to those achieved in the treatment of other malignancies.

## 5. Conclusions

This study confirms that complete surgical resection remains the decisive factor for survival in paediatric hepatocellular carcinoma, including both conventional HCC and fibrolamellar carcinoma. While upfront surgery should be pursued whenever feasible, our findings show that neoadjuvant chemotherapy can achieve secondary resectability in a subset of initially unresectable cHCC cases, providing a valuable treatment option. In contrast, FLC demonstrates minimal to no chemosensitivity, underscoring the urgent need for more effective systemic therapies tailored to its distinct biology.

Given the differential responses by histologic subtype, these results support histology-specific therapeutic strategies and reinforce the importance of early, aggressive surgical evaluation.

## Figures and Tables

**Table 1:** Demographics of the whole cohort.

	No. of Patients (%)					
Variable	Total, N = 43	Conventional HCC, N = 27	FLC, N = 16	*p* (HCC vs. FLC)	Age (Mean ± SD, mo.)	*p*
Age (mean ± SD, mo.)	158.1 ± 46.0	143.6 ± 50.1	182.5 ± 23.7	**0.007**		
Underlying disease				**0.013**		**0.029**
Yes	8 (18.6)	8 (29.6)	0 (0)		118.0 ± 61.1	
No	32 (74.4)	16 (59.3)	16 (100)		168.8 ± 38.0	
Unknown	3 (7.0)	3 (11.1)	0 (0)			
AFP at diagnosis, ng/mL				**<0.0001**		
<100	22 (51.2)	7 (25.9)	15 (93.8)			
≥100	20 (46.5)	20 (74.1)	0 (0)			
Unknown	1 (2.3)	0 (0)	1 (6.2)			
PRETEXT				0.100		>0.999
I	6 (14.0)	3 (11.1)	3 (18.8)		150.8 ± 52.1	
II	13 (30.2)	5 (18.5)	8 (50.0)		179.5 ± 22.7	
III	18 (41.8)	14 (51.9)	4 (25.0)		151.3 ± 52.8	
IV	6 (14.0)	5 (18.5)	1 (6.2)		139.5 ± 50.0	
Distant metastasis				0.343		**0.043**
Yes	17 (39.5)	9 (33.3)	8 (50.0)		172.0 ± 50.4	
No	26 (60.5)	18 (66.7)	8 (50.0)		149.0 ± 41.3	

Abbreviations: HCC, hepatocellular carcinoma; FLC, fibrolamellar carcinoma; HCN-NOS, hepatocellular neoplasm, not otherwise specified; SD, standard deviation; mo., months; PRETEXT, pretreatment extent of disease. bold: significant values.

**Table 2:** Treatment and outcomes.

	No. of Patients (%)																
		Histology			Age		Comorbidity			PRETEXT					Distant Metastases		
Variable	Total, N = 43	Conventional HCC, N = 27	FLC, N = 16	*p*	Mean ± SD (mo.)	*p*	No = 32	Yes = 8	*p*	I (6)	II (19)	III (12)	IV (6)	*p*	Yes = 17	No = 26	*p*
biopsy				>0.99		0.61			**<0.01**					0.64			0.27
Yes	34 (79.1)	21 (77.8)	13 (81.3)		13.49 ± 3.28		30 (88.2)	3 (37.5)		4 (66.7)	15 (78.9)	9 (75)	6 (100)		15 (88.2)	19 (73.1)	
No	9 (20.9)	6 (22.2)	3 (18.7)		12.00 ± 5.55		4 (11.8)	5 (62.5)		2 (33.3)	4 (21.1)	3 (25)	0		2 (11.8)	7 (26.9)	
systemic therapy																	
CTX *neoadj.*	34 (79.1) *21 (48.9)*	21 (77.8) *16 (59.3)*	13 (81.3) *5 (31.3)*	0.69	Y:13.63 ± 3.45 N:11.12 ± 5.11	0.18	31 (96.9)	3 (37.5)	**<0.01**	5 (83.3)	14 (73.7)	10 (83.3)	6 (100)	0.70	15 (88.2)	19 (73.1)	0.13
Sorafenib *neoadj.*	29 (67.4) *16 (37.2)*	16 (59.3) *10 (37.0)*	13 (81.3) *6 (37.5)*	0.19	Y:14.15 ± 3.31 N:11.18 ± 4.19	**0.02**	28 (87.5)	1 (12.5)	**<0.01**	3 (50)	12 (60)	10 (83.3)	4 (66.7)	0.50	16 (94.1)	12 (46.2)	**<0.01**
Antibodies	7 (16.3)	2 (7.4)	4 (25)														
surgery				>0.99		0.25			0.60					0.14			**0.01**
no OP	7 (16.3)	4 (14.8)	3 (18.7)		14.50 ± 4.62		5 (14.7)	2 (25)		1 (16.7)	2 (10.5)	1 (8.3)	3 (50.0)		6 (35.3)	1 (3.8)	
part. hepatectomy	29 (67.4)	17 (63.0)	12 (75)		13.38 ± 3.29		26 (76.5)	2 (25)	}**0.01**	5 (83.3)	15 (79.0)	9 (75)	0	}**0.01**	10 (58.8)	19 (73.1)	}0.40
OLT	7 (16.3)	6 (22.2)	1 (6.3)		11.06 ± 4.86		3 (8.8)	4 (50)		0	2 (10.5)	2 (16.7)	3 (50.0)		1 (5.9)	6 (23.1)	
resection				0.46		0.25			>0.99					0.37			**<0.01**
Non-complete	10: 7 no OP, 3 incomplete	5 (18.5)	5 (31.3)		12.91 ± 3.54		8 (23.5)	2 (25)		1 (16.7)	3 (15.8)	3 (25)	3 (50)		9 (52.9)	1 (3.8)	
complete (R0)	33: 17 primary (3x OLT) 16 delayed (4x OLT)	22 (81.5)	11 (68.7)		14.07 ± 4.80		26 (76.5)	6 (75)		5 (83.3)	16 (84.2)	9 (75)	3 (50)		8 (47.1)	25 (96.2)	

Abbreviations: FLC, fibrolamellar carcinoma; SD, standard deviation; mo., months; PRETEXT, pretreatment extent of disease; CTx, chemotherapy; neoadj., neoadjuvant; OP, operation; part., partial; OLT, orthotopic liver transplantation; R0, complete resection with microscopically negative margins. bold: significant values.

**Table 3:** Hazard ratio (Mantel–Haenszel) for death.

Variable	Group	HR (95% CI)
Histology	cHCC	Reference
	FLC	1.64 (0.57, 4.72)
AFP at Dx, ng/mL	<100	Reference
	≥100	0.74 (0.27, 2.05)
PRETEXT Group	I, II, III	Reference
	IV	7.06 (1.27, 39.34)
Distant metastasis	No	Reference
	Yes	5.53 (1.91, 16.02)
Extent of surgery	R0	Reference
	R1, R2, non-resected	10.44 (2.59, 42.09)
Timing of surgical	at diagnosis	Reference
resection	after chemotherapy	0.58 (0.17, 2.04)

Abbreviations: HR, hazard ratio; CI, confidence interval; cHCC, conventional hepatocellular carcinoma; FLC, fibrolamellar carcinoma; AFP, Alpha-1-Fetoprotein; Dx, diagnosis; PRETEXT, pretreatment extent of disease.

**Table 4:** Patients without primary tumour resection (resection at diagnosis).

UPN	Age (y)	Histo-Logy	Local Extent, PRETEXT Stage; Vasc. Involvement; Rupture	Dist. Metastasis or Extrahepatic Disease	Indication or Reason for CTx Instead of Primary Resection?	Prim. Resect-Ability by Part. Hep./OLT?	Neoadjuvant CTx	Sora-Fenib	Response (RECIST)	Extent of Surgery (Ever Disease Free?)	Adjuvant CTx	Survival After Dgn.(mo.)
18	14	FLC	Unifocal, III; V-, P-; R-	**E+** (diaphragm), M-	extrahepatic disease	no	1xPLADO, 2xPLADO+S	Yes	SD	R0 (part. hep.)	2xGEMOX 2xPLADO	13.4 (CR)
46	10	HCC	Unifocal, I; V-, P-; **R+**	E-, M-	tumour rupture	yes (part. hep.)	1xTACE (Doxo)	No	n.a.	R0 (part. hep.)	None	61.7 (dod)
69	10	HCC	**Multifocal**, III; V-, P-; R-	E-, **M+ (lung)**	distant metastases (+multi- focality)	no	1xPLADO, 3xPLADO+S+	Yes	PR	R0 (part. hep.)	2xPLADO+S Sora 1a	45.8 (CR)
79	9	HCC	**Multifocal**, **IV**; V-, P-; R-	E-, **M+ (lung)**	distant metastases, PRETEXT IV (+multifocality)	no	SIOPEL 4 (Cisplatin, Carbo, Doxo)	No	PR	R0 (OLT)	PLADO, Carbo+ Doxo, Sora maint	17.5 (dod)
108	17	FLC	Unifocal, I; V/P n.a.; **R+**	**E+**, **M+ (peritoneum)**	extrahepatic disease with metastasis	no	1xPEI, 1xGEMOX+S	Yes	n.a.	None	n.a.	1.61 (dod)
116	5	HCC	**Multifocal**, **IV**; V n.a., **P+**; R-	E-, **M+ (lung)**	distant metastases, PRETEXT IV	No	4xPLADO/10xRIST	No	PD (after 4xPLADO)	None	n.a.	14.55 (dod)
130	10	HCC	**Multifocal**, **IV**; V-, **P+**; R-	E-, M-	PRETEXT IV, P+ (+multifocality)	yes (OLT)	SIOPEL 3HR, (3xPLADO/Doxo,1xCis)	No	SD	R0 (OLT)	None	49.5 (CR)
136	9	HCC	**Multifocal**, II; V-, P-; **R+**	E-, M-	tumour rupture (+multifocality)	yes (part. hep.)	Cis, 3xPLADO+S	Yes	PR	R0 (part. hep.)	2xPLADO+S Sora maint.	122.3 (CR)
147	18	FLC	Unifocal, II; V-, P-; **R+**	E-, **M+ (peritoneum)**	Metastasis	no	2xPLADO+S, 1xPLADO+ Erlonitib+Bevacizumab	Yes	PD (mets)/ PR (liver)	None	n.a.	15.93 (dod)
169	11	HCC	Unifocal, II; V-, P-; R-	E-, M-	missing reference evaluation	yes (part. hep.)	1xPLADO+S	Yes	PR	R0 (part. hep.)	None	4.7 (doc)
200	17	FLC	Unifocal, I; V-, P-; R-	**E+ (lymph nodes)**, M-	tumour extent (+extrahepatic disease)	no	1xPLADO+S	Yes	SD	R0 (part. hep.)	2xPLADO+S	111.1 (rel.)
229	14	HCC	**Multifocal**, III; V-, **P+**; **R+**	**E+**, M-	ingrowth portal vein, rupture, (+multifocality, +extrahepatic disease)	no	6xPLADO+Doxo, 1xCis	No	PR	R0 (part. hep.)	1xPLADO+Doxo 1xToptecan+Doxo	105.8 (rel.)
251	17	HCC	**Multifocal**, **IV**; V-, P-; R (n.a.)	**E (n.a.)**, **M+ (lung)**	distant metastases, PRETEXT IV	no	1xPLADO+S, 3xAtezolizumab+Bevazizumab, 3xGEMOX	Yes	PD (all therapies)	None	n.a.	10.45 (dod)
283	13	HCC	**Multifocal**, III,; V-, P-; **R+**	E-, M-	tumour rupture (+multifocality)	yes (part. hep.)	2xTACE, 3xPLADO	No	PR	R0 (part. hep.)	3xPLADO	92.2 (CR)
294	7	HCC	Unifocal (central), III; V-, P-; R-	E-, M-	central localization	yes (**OLT**)	2xPLADO, 2xPLADO+S	Yes	PR	R0 (part. hep.)	2xPLADO+S Sora maint.	34.6 (dod)
301	15	HCC	Unifocal (central), III; V-, P-; R-	E-, M-	central localization	yes (OLT)	1xPLADO, 2xPLADO+S 1xGEMOX+S	Yes	SD	R0 (OLT)	None	49.22 (CR)
304	12	HCC	**Multifocal**, **IV**; **V+**, P n.a.; R-	E-, **M+ (lung, peritoneum)**	distant metastases, PRETEXT IV	no	2xPLADO+S	Yes	PR	None	n.a.	7.52 (death, n.a.)
360	17	HCC	Unifocal (central), II; n.a.; R-	E-, **M+ (omentum, lung)**	distant metastases, central localization	no	6xPLADO+S	Yes	PR	R0 (part. hep.)	2xPLADO+S Sora maint.	24.05 (dod)
381	13	HCC	**Multifocal**, II; V/P n.a.; R-	E-, M-	no chemotherapy, watch and wait	yes (part. hep.)	None	No	SD	None	n.a.	29.04 (SD)
404	16	HCC	**Multifocal**, II; V-, P-, R-	E-, **M+ (ribs)**	distant metastases (+multi- focality)	no	2xPLADO+S 2xCarbo/Doxo+S	Yes	SD	R0 (part. hep.)	1xCarbo/Doxo+S, 1xCarbo+S	143.11 (rel.)
436	16	FLC	Unifocal, III; V/P n.a.; R-	E-, **M+ (lung)**	distant metastases	no	No CTx, S, Lenvatinib, Nivolumab, Pembrolizumab	Yes	PR (mets)/PD (liver)	None (only SIRT)	n.a.	68.80 (PD)
439	13	FLC	**Multifocal**, **IV**; V-, P-; R-	**E+ (lymph node)**, M-	PRETEXT IV	no	1xPLADO+S	Yes	SD	R0 (OLT)	2xGEMOX+S 1xPLADO+S, 1xS	61.57 (rel., CR)
481	12	HCC	Unifocal, II; V-, P-; R-	E-, M-	tumour extent	yes (OLT)	4xPLADO+S	Yes	PR	R0 (part. hep.)	2xPLADO+S Sora maint.	99.88 (CR)

Abbreviations: UPN, unique patient number; y, years; vasc., vascular; PRETEXT, pretreatment extent of disease; CTx, chemotherapy; part. hep., partial hepatectomy; OLT, orthotopic liver transplantation; RECIST, response evaluation criteria in solid tumours; mo., months; dgn., diagnosis; n/a, not applicable or not available; V, ingrowth vena cava/all hepatic veins; P, ingrowth both R and L portal veins or bifurcation; R, tumour rupture prior to diagnosis; E, contiguous extrahepatic tumour; M, distant metastases; IVC, inferior vena cava; PLADO, cisplatin/doxorubin; S, Sorafenib; Sora maint., Sorafenib maintenance therapy; Doxo, Doxorubicin; GEMOX, Gemcitabine/oxaliplatin; TACE, transarterial chemoembolization; SIRT, selective internal radiation therapy; PR, partial response; PD, progressive disease; SD, stable disease; CR, complete remission; res., resection; dod, death of disease; doc, death of operative complication; rel., relapse. Bold：Positive annotations which contributed to secondary resection.

## Data Availability

The data presented in this study are not publicly available due to privacy and ethical restrictions.

## Abbreviations

The following abbreviations are used in this manuscript:

AFP	Alpha-fetoprotein
cHCC	Conventional Hepatocellular Carcinoma
CR	Complete Remission
CTX	Chemotherapy
FLC	Fibrolamellar Carcinoma
HCC	Hepatocellular Carcinoma
MRI	Magnetic Resonance Imaging
OLT	Orthotopic Liver Transplantation
OS	Overall Survival
PD	Progressive Disease
PLADO	Cisplatin and Doxorubicin
PR	Partial Remission
PRETEXT	Pretreatment Extent of Disease
R0	Complete (Microscopically Negative) Resection
SD	Stable Disease
SIOPEL	International Society of Pediatric Oncology Liver Tumour Study Group
SIRT	Selective Internal Radiation Therapy
UPN	Unique Patient Number
